# Routine Post-operative Radiographs Following Hip Hemiarthroplasty: Is It a Necessity?

**DOI:** 10.7759/cureus.19049

**Published:** 2021-10-26

**Authors:** Kavyansh Bhan, Kamrul Hasan, Basharat Ghafoor Khan, Hassan Shafiq, Mahesh Pimplé

**Affiliations:** 1 Trauma and Orthopaedics, Barts Health NHS Trust, London, GBR

**Keywords:** geriatric hip fracture, xray, partial hip arthroplasty, bipolar hemiarthroplasty, fracture neck of femur

## Abstract

Femoral neck fractures are one of the most common fractures treated by an Orthopaedic surgeon. Arthroplasty is the recommended management for intracapsular neck of femur fractures in the elderly population owing to the high risk of avascular necrosis of the femoral head following an internal fixation. Elderly patients with intracapsular fractures deemed high risk for anaesthesia (American Society of Anaesthesiology Grade more than 2) are recommended a hip hemiarthroplasty. Routine practice throughout the United Kingdom is to obtain a postoperative check radiograph for all hip hemiarthroplasty patients prior to their discharge from the hospital. This may be done for various reasons like checking the presence of any peri-prosthetic fracture, the position of the components along with the presence of any dislocation. However, it is unclear whether a radiograph is the sole identifier of such complications. Through this study, we aim to analyse whether routine recommendation of post-operative radiographs following hip hemiarthroplasty affects the clinical outcome, and whether it is effective in identifying potential complications before the patients report any signs or symptoms.

## Introduction

With an annual incidence of 1.6 million fractures, estimated to rise to more than 7 million by 2050, femoral neck fractures are one of the most common fractures treated by an orthopaedic surgeon [1,2]. Seen more commonly in the older population, these types of fractures have high morbidity and mortality associated with them. Arthroplasty is the recommended management for the intracapsular neck of femur fractures in this subset of the population owing to the high risk of avascular necrosis of the femoral head following an internal fixation [3]. The choice between a total hip replacement (THR) and hip hemiarthroplasty (HA) for the treatment of these fractures continues to be a hotly debated topic. The National Institute of Health and Care Excellence (NICE) in the United Kingdom (UK) has laid down clear guidelines regarding surgical treatment of intracapsular femoral neck fractures. They have recommended that THR be offered to all patients who are able to independently mobilise outdoors with no more than the use of a stick and have no diagnosed cognitive impairment [4]. For other elderly patients, and patients deemed high risk for anaesthesia (American Society of Anaesthesiology Grade more than 2), it is recommended that a HA be offered [4]. Routine practice throughout the UK is to obtain a postoperative check radiograph for all HA patients prior to their discharge from the hospital [5]. This may be done for various reasons like checking the presence of any peri-prosthetic fracture, the position of the components along with the presence of any dislocation. However, whether a post-operative radiograph is the sole identifier of these complications is unclear [6]. Most of the patients may present with associated symptoms which may point towards the presence of a complication even before the completion of a post-operative radiograph. Hence, the benefit of routine post-operative radiographs following HA is still unvalidated [6]. Through this study, we aim to analyze whether routine recommendation of post-operative radiographs following HA affects the clinical outcome, and whether it is effective in identifying potential complications which may be made worse by mobilisation before the patient reports any symptoms. This study was conducted at a District General Hospital (DGH) in the Greater London region which is a frailty hip fracture centre and accepts patients with hip fractures from a catchment population of more than 2.6 million.

## Materials and methods

A retrospective chart review of consecutive patients undergoing hip hemiarthroplasty for intracapsular neck of femur fractures from January 2021 to June 2021 was carried out at our institution.

Inclusion criteria

Patient records were reviewed using the internal Trust Trauma Database. All patients who underwent hip hemiarthroplasty for intracapsular neck of femur fractures were included. All patients included had a minimum of one postoperative radiograph of the pelvis. The time to check the radiograph after the surgery was also evaluated. A note was made if the patient underwent multiple check radiographs. All radiographs for patients done up to 90 days after the day of surgery were included and reviewed. Only those patients were included who had at least one post-operative radiograph done in the hospital before discharge. 

Exclusion criteria 

All patients who underwent hip hemiarthroplasty for reasons other than a traumatic neck of femur fracture were excluded from the study. This included patients who had failed internal fixation and underwent hemiarthroplasty for the same reason. All patients who had polytrauma or more than one concurrent fracture were excluded from the study. Patients who did not have at least one postoperative radiograph of the pelvis were excluded. Patients who did not have a postoperative radiograph in the hospital prior to their discharge were not included. Any patient who underwent internal fixation for intracapsular neck of femur fracture was also excluded. Patients who underwent a total hip arthroplasty were also not included in the study. Any patient who did not have an operation note documenting the details of the surgery was excluded. 

Methods

A total of 125 eligible records were included in the study. The Picture Archiving and Communication System (PACS) was used to review the radiographs of the eligible patients. All 125 patients had a postoperative radiograph. As per hospital policy, all patients had one postoperative anteroposterior (AP) radiograph of the pelvis and one lateral (Lat) radiograph of the operated hip in the same sitting. All the radiographs were taken prior to the discharge of the patient from the hospital. The immediate post-operative period was defined as the first 5 days after surgery. The radiographs were reviewed to establish the presence of any mechanical complications like a dislocation or fracture which was evident on the radiograph. All radiographs done prior to the discharge of the patient were reviewed. If the patients had any repeat radiographs in the 90 days following the surgery, they were also reviewed to establish the presence of any mechanical complications including fracture and dislocation. The operation notes were also reviewed and variables like seniority of the operating surgeon, approach used for the surgery and any intra-operative complications were noted. The nature of hemiarthroplasty, viz. cemented or uncemented, was also noted.

## Results

In the study period of 6 months, 133 patients underwent hip hemiarthroplasty at our institute. This cohort comprised 86 females and 47 males aged 60 years - 99 years with an average age of 85.43 years. Eight patients had no post-operative radiograph on the PACS system of the hospital. These eight patients had clinically deteriorated following the surgery and were put on an end-of-life care pathway, obviating the need for radiographs. The final study group thus consisted of 125 patients, all of whom had post-operative radiographs. Three patients had subsequent repeat radiographs within 90 days of their discharge from the hospital owing to clinical suspicion of fracture or dislocation.

**Figure 1:**
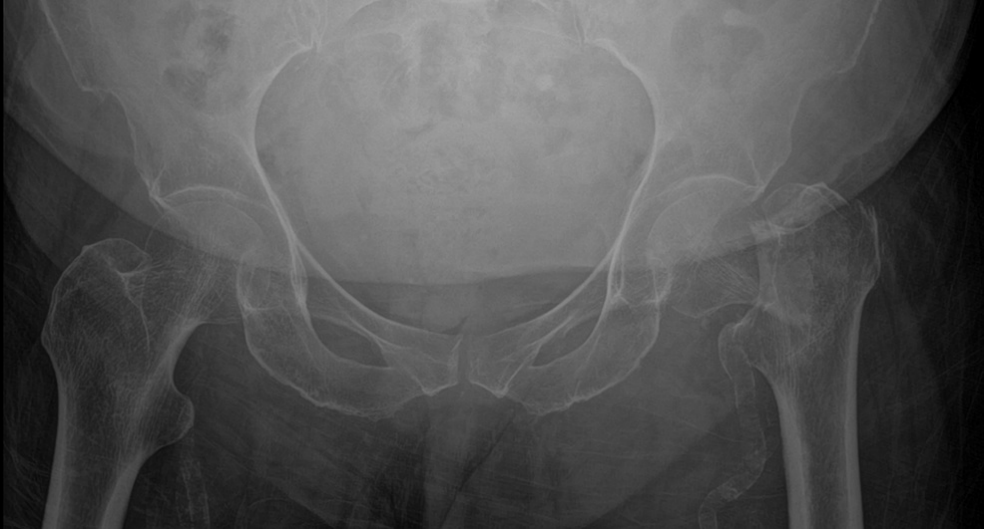
Pre-operative radiograph of the only patient in the study who subsequently had a prosthetic dislocation

**Figure 2:**
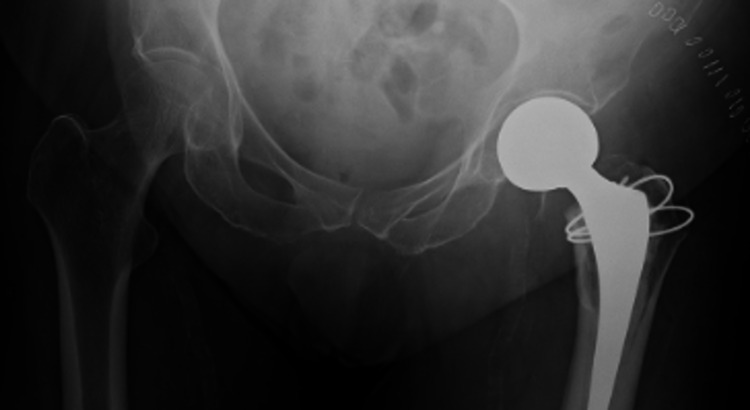
Immediate postoperative radiograph of the patient who subsequently had a prosthetic dislocation This radiograph demonstrates an acceptable prosthesis placement and no concern was raised which would have suggested a future prosthesis failure.

**Figure 3:**
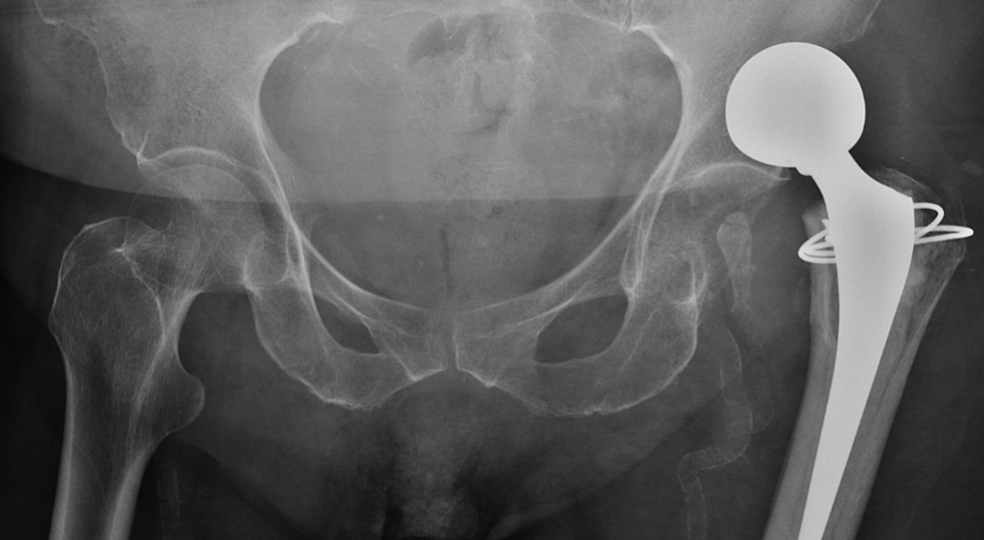
Postoperative dadiograph at day 35 demonstrating a dislocation of the bipolar hip hemiarthroplasty prosthesis

Of the 125 hemiarthroplasties, immediate postoperative dislocation was not noted in any patient. All 125 immediate postoperative radiographs were deemed to be satisfactory. Subsequent mechanical complications were noted in one patient following a satisfactory initial postoperative radiograph (1/125 = 0.80%). The patient in question was found to have a dislocation on the subsequent check radiograph (Figures 1-3). Clinical suspicion was raised prior to the repeat radiograph with the patient complaining of pain and inability to weight bear on the affected side. 

On review of the operation notes, it was noted that all the surgeries were performed through a direct lateral approach with the patient in a lateral position as described in a recent paper published by Petis et al [7]. The surgeries were performed by surgeons of varying experiences, ranging from second-year trauma and orthopaedic trainees to specialist hip and knee consultants. Both cemented and uncemented hemiarthroplasty were performed taking into account the patient’s comorbidities, anaesthetist recommendation and surgeon preference. All the surgeries involved the use of bipolar prosthesis.

The average time to check the radiograph was 3.02 days (range 1-8 days). It is estimated that a single postoperative check radiograph costs approximately £53 per patient [6]. This would mean that the radiographs for patients in this study alone cost the hospital around £6,625. A delay in obtaining the check radiograph, however, did not affect the rehabilitation of patients. The patients were routinely allowed to weight-bear as tolerated before a check radiograph.

## Discussion

Postoperative radiographs following hip hemiarthroplasty have been long used as a screening measure to identify any major mechanical complications. However, the efficacy of a screening test is dependent on the prevalence of the condition being screened, along with its sensitivity and specificity [8]. Its usefulness is measured by its impact on patient care and clinical outcome. Through this study, it was found that the incidence of abnormal postoperative radiographs was extremely low. While no abnormal radiographs were identified in the immediate postoperative period, only 0.80% of the radiographs detected a mechanical complication in the 90 days following the surgery. This indicates that postoperative imaging for HA patients may not play any significant role in shaping the postoperative care of these patients. Moreover, NICE recommends the mobilisation of HA patients by the first postoperative day to avoid developing complications that may be associated with prolonged bed rest [9]. Hence, getting radiographs for all postoperative HA patients may be unnecessary since patients with mechanical complications who do need a postoperative radiograph are very likely to exhibit signs and symptoms associated with the complications [9].

Interestingly, a questionnaire survey of 450 British trauma and orthopaedic consultants published by Chakravarthy et al. discussed the potential indications for getting postoperative radiographs for HA patients [9]. They noted that many consultants ordered postoperative radiographs for medico-legal reasons rather than a clinical indication [9]. However, a review of the guidelines for doctors laid down by the Royal College of Radiologists indicates that any investigation requested should be mandatorily justified by its clinical need and a possibility to affect further management of the patient [10]. Hence, exposing patients to radiation from postoperative radiographs without a clinical indication may not be justifiable. Another retrospective chart review evaluating the efficacy of check radiographs for HA patients published recently found that unless there was a significant complication noted in the history or physical examination of HA patients, an abnormal radiograph alone was unlikely to alter the course of treatment for patients [11]. Similar findings are echoed in the study published by Maling et al. too [6]. This may imply that check radiographs alone have questionable significance and utilising them as a screening tool in the absence of any concerning symptoms or signs may be a futile exercise.

Owing to the COVID-19 pandemic, many hospitals have now started changing their routine practice to allow optimum use of the limited resources, both financial and human. Postoperative radiograph of an inpatient is a resource-intense procedure that involves the utilisation of services of various human resources, ranging from a porter to the radiographer. The patient may also encounter multiple people while being shifted for a radiograph, thereby increasing the risk of transmission of COVID-19. Hence, various hospitals across the United Kingdom have now identified this and the rate of routine post-operative radiographs following hip hemiarthroplasties has shrunk from 98% before the pandemic to 74% during the pandemic [12]. 

There is sufficient evidence in the literature to suggest that routine postoperative radiographs in orthopaedic patients seldom affect the clinical outcome or management of the patients [13-15]. Although most of these studies have evaluated patients who underwent surgeries like total knee arthroplasty, total hip arthroplasty or spinal surgeries, we found similar results in patients being managed with hip hemiarthroplasty. To date, there is limited data on the utility of routine postoperative radiographs for hip hemiarthroplasty and this study is one of the few studies which has addressed this topic. 

The primary limitation of this study is the small sample size. Large multi-centre studies may be needed to reaffirm our findings before any strong recommendation can be made on the basis of this study. Although current evidence supports the judicial use of radiographs and suggests ceasing routine screening postoperative radiographs, further studies are needed to examine the impact of cessation of routine radiographs and its effects on patient safety. 

## Conclusions

Our study demonstrates minimal clinical benefit, high costs and a potential safety and radiation hazard from routine postoperative radiographs following a hip hemiarthroplasty. Thus, the authors recommend postoperative radiographs for hip hemiarthroplasty patients only if clinically indicated by signs and symptoms like pain, inability to weight-bear or potential infection.
